# DOA Estimation Based on Real-Valued Cross Correlation Matrix of Coprime Arrays

**DOI:** 10.3390/s17030638

**Published:** 2017-03-20

**Authors:** Jianfeng Li, Feng Wang, Defu Jiang

**Affiliations:** Array and information processing laboratory, College of computer and information, Hohai University, Nanjing 211100, China; wangfeng@hhu.edu.cn (F.W.); jiangdefu@hhu.edu.cn (D.J.)

**Keywords:** direction of arrival estimation, coprime arrays, cross-correlation matrix, pair matching

## Abstract

A fast direction of arrival (DOA) estimation method using a real-valued cross-correlation matrix (CCM) of coprime subarrays is proposed. Firstly, real-valued CCM with extended aperture is constructed to obtain the signal subspaces corresponding to the two subarrays. By analysing the relationship between the two subspaces, DOA estimations from the two subarrays are simultaneously obtained with automatic pairing. Finally, unique DOA is determined based on the common results from the two subarrays. Compared to partial spectral search (PSS) method and estimation of signal parameter via rotational invariance (ESPRIT) based method for coprime arrays, the proposed algorithm has lower complexity but achieves better DOA estimation performance and handles more sources. Simulation results verify the effectiveness of the approach.

## 1. Introduction

Direction of arrival (DOA) estimation via a sensor array is an important issue for radar, sonar, and wireless communication systems [1,2,3,4], and many well-known DOA estimation algorithms have been established [5,6,7,8,9,10,11,12]. Multiple signal classification (MUSIC)-based methods [5,6] obtain the DOA estimation via a peak search, which is highly computational. To reduce the complexity, root-MUSIC based methods [7,8] were proposed to estimate the DOA via polynomial root finding instead of the peak search. Estimation of signal parameters via the rotational invariance technique (ESPRIT)-based methods [9,10] exploit the invariance property within the signal subspace to obtain closed-form DOA solutions, and generalized ESPRIT [11] can be suitable for arbitrary geometries. The support vector classifier-based method proposed in [12] can obtain DOA estimation with a low complexity by exploiting a multi-scaling procedure. These methods all can provide accurate DOA estimations by using arrays whose inter-element distances are no larger than a half-wavelength to avoid the ambiguity problem of angle estimation. However, the compact arrays they used have many limitations, e.g., the high estimation error bound and the mutual coupling problem [13,14].

As a new concept for array geometry, coprime arrays use two sparse uniform linear arrays (ULAs) with coprime antenna numbers and coprime inter-element distances to achieve high resolution DOA estimation and reduce mutual coupling influence [15,16,17]. To overcome the ambiguity problem of DOA estimation using sparse arrays, the common peaks of the MUSIC spectra obtained from the two coprime subarrays are selected to uniquely determine the DOA estimations [18], but the high-computational peak searches are required. Thereafter, in order to reduce the complexity involved in the peak search of whole angular space, a partial spectral search (PSS) MUSIC method was proposed in [19] to reduce the search range, and an ESPRIT-based method was proposed in [20] to estimate the DOA without peak search. However, these methods all process the subarrays separately, and the obtained results from the two subarrays are mis-pairing, which result in angular interference between different sources and degradation of the DOA estimation performance, especially with low signal to noise ratio (SNR). To deal with this problem, Ref. [20] proposed an additional processing procedure relying on peak search and angle difference chosen, but this makes the algorithm inefficient.

In this paper, a low-complexity DOA estimation method using real-valued cross correlation matrix (CCM) of coprime arrays is proposed. Based on the uniformity of the subarrays, the smaller aperture between the two coprime subarrays can be extended and real-valued CCM can be constructed. After singular value decomposition (SVD) of the real-valued CCM, the relationship between the two subspaces of the subarrays is analysed, and then the closed-form solutions of the DOA estimations corresponding to the two subarrays can be simultaneously obtained with automatically pairing. The automatically paired solutions make it simple and accurate to determine the unique DOA estimation. Compared to the PSS method in [19] and ESPRIT-based method in [20], the proposed algorithm requires lower complexity, achieves better DOA estimation performance and handles more sources. Multiple simulations are conducted to verify the effectiveness of our approach.

**Notation 1.** ${\left(.\right)}^{T}$, ${\left(.\right)}^{*}$, ${\left(.\right)}^{H}$, ${\left(.\right)}^{-1}$*, and* ${\left(.\right)}^{+}$*denote the operations of transpose, conjugate, conjugate-transpose, inverse, and pseudo-inverse, respectively.*E[.]*is the expectation operator. Re (.) and Im (.) represent the real and imaginary parts of the complex, respectively.*

## 2. Data Model

**Figure 1:**
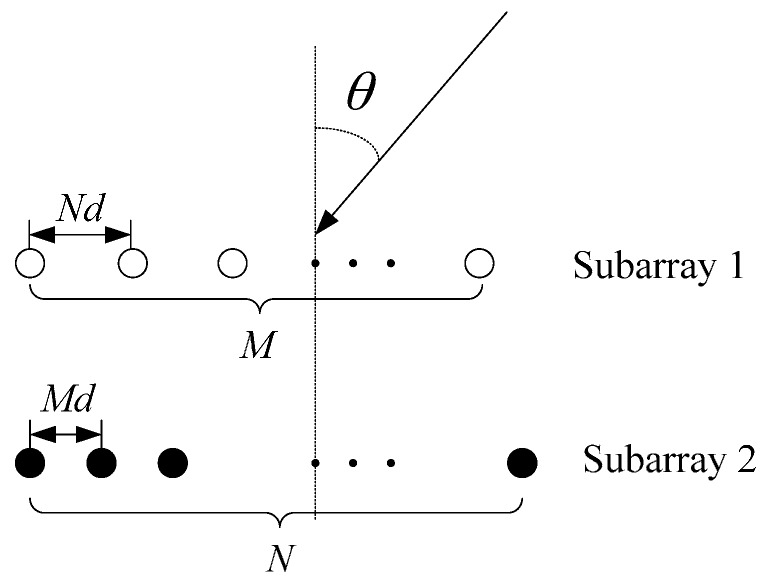
The structure of coprime arrays.

As Figure 1 shows, coprime arrays consist of two sparse ULAs, where subarray 1 has *M* elements with *Nd* being the inter-element spacing and subarray 2 has *N* elements with *Md* being the inter-element spacing. *M* and *N* are coprime integers, and *d* is generally set as d=λ/2, where *λ* denotes the signal wavelength.

Assume that there are *K* far-field uncorrelated signals impinging on the arrays, and use $\theta_{k}$ to denote the DOA of the *k*th signal with respect to the array normal, then the outputs of the two subarrays are:

(1a) $$x_{1}(t)=A_{1}s(t)+n_{1}(t)$$

(1b) $$x_{2}(t)=A_{2}s(t)+n_{2}(t)$$

where $s(t)=[s_{1}(t),...,$ $s_{K}(t){]}^{T}$ is the baseband signal vector. $n_{1}(t)$ and $n_{2}(t)$ are the noise vectors, which are assumed to be uncorrelated to each other and independent to the signals. $A_{1}=[a_{1}(\theta_{1}),...,a_{1}(\theta_{K})]$ and $A_{2}=[a_{2}(\theta_{1}),...,a_{2}(\theta_{K})]$ are the direction matrices of subarray 1 and subarray 2, respectively. The steering vectors corresponding to the *k*-th signal are:

(2a) $$a_{1}(\theta_{k})\;=\;{\left[1,e^{-jN\pi \sin\theta_{k}},...,e^{-j(M-1)N\pi \sin\theta_{k}}\right]}^{T}$$

(2b) $$a_{2}(\theta_{k})\;=\;{\left[1,e^{-jM\pi \sin\theta_{k}},...,e^{-j(N-1)M\pi \sin\theta_{k}}\right]}^{T}$$

## 3. Proposed DOA Estimation Method

In this section, the proposed DOA estimation method will be presented. Section 3.1, Section 3.2 and Section 3.3 contain the three major steps of the method, and Section 3.4 includes the algorithm summaries, algorithm comparison, and remarks.

### 3.1. Construction of Real-Valued CCM with Extended Aperture

In this section, we will construct a real-valued CCM with extended aperture. As the maximum number of detectable source depends on the minimum aperture of the two subarrays, we consider extending the smaller aperture between the two subarrays. In the following section, we assume subarray 1 has smaller aperture than subarray 2, i.e., *M* < *N* (refer to remark 1 for the situation when *M* > *N*). 

Realize that the subarrays are sparse, but still uniform, which means that their manifolds are symmetrical and do not exhibit inclination [21]. Define unitary matrices as:

(3a) $$Q_{2k}=\frac{1}{\sqrt{2}}\left[\begin{array}{cc} I_{k} & jI_{k}\\ \Pi_{k} & -j\Pi_{k} \end{array}\right]$$

(3b) $$Q_{2k+1}=\frac{1}{\sqrt{2}}\left[\begin{array}{ccc} I_{k} & 0 & jI_{k}\\ 0 & \sqrt{2} & 0\\ \Pi_{k} & 0 & -j\Pi_{k} \end{array}\right]$$

where $I_{K}$ and $\Pi_{K}$ are *K* × *K* identity matrix and reverse identity matrix (90° rotation of $I_{K}$), respectively. Assume *N* is odd, then the steering vector of subarray 2 is transformed as:

(4) $$\begin{array}{l} a_{2r}(\theta_{k})=\;Q_{N}^{H}a_{2}(\theta_{k})\\ =\;e^{\left(-j\frac{\left(N-1\right)}{2}M\pi \sin\theta_{k}\right)}\sqrt{2}\times [\cos(\frac{\left(N-1\right)}{2}M\pi \sin\theta_{k}),\cdots \cos(\frac{\left(N-3\right)}{2}M\pi \sin\theta_{k}),\cos(M\pi \sin\theta_{k}),\\ 1/\sqrt{2},\sin(\frac{N-1}{2}M\pi \sin\theta_{k}),\cdots \sin(M\pi \sin\theta_{k}){]}^{T}\\ =v_{k}h_{2}(\theta_{k}) \end{array}$$

where $v_{k}\;=\;e^{\left(-j\frac{\left(N-1\right)}{2}M\pi \sin\theta_{k}\right)}$ is a scalar. According to Equation (4), the steering vector can be transformed into a real-valued vector $h_{2}(\theta_{k})$ multiplied by a scalar $v_{k}$.

Construct the CCM of the two outputs in Equation (1) as:

(5) $$R_{c}=E[x_{1}(t)x_{2}^{H}(t)]=A_{1}R_{s}A_{2}^{H}$$

where $R_{s}=\;diag(\sigma_{1}^{2},...,\sigma_{K}^{2})$ is a real-valued diagonal matrix composed of signal powers [22]. According to Equation (4), transform CCM in Equation (5) as:

(6) $$\begin{array}{cc} R_{cr} & =\;R_{c}Q_{N}=A_{1}R_{s}A_{2}^{H}Q_{N}\\ & =A_{1}R_{s}{\Psi_{v}}^{*}{H_{2}}^{T}=\;A_{1}{\Psi_{v}}^{*}R_{s}{H_{2}}^{T} \end{array}$$

where $H_{2}=\;[h_{2}(\theta_{1}),...,h_{2}(\theta_{K})]$ is the real-valued direction matrix of subarray 2, and $\Psi_{v}=\;diag(v_{1},...,v_{K})$ is a diagonal matrix, which means the orders of ${\Psi_{v}}^{*}$ and $R_{s}$ can be exchanged (has been shown in Equation (6)). Based on the real-valued property of $H_{2}$, construct extended CCM as:

(7) $$R_{z}=\left[\begin{array}{c} \Pi_{M}R_{cr}\\ {R_{cr}}^{*} \end{array}\right]=\left[\begin{array}{c} \Pi_{M}A_{1}{\Psi_{v}}^{*}R_{s}{H_{2}}^{T}\\ {A_{1}}^{*}\Psi_{v}R_{s}{H_{2}}^{T} \end{array}\right]=\underset{A_{1E}}{\underset{︸}{\left[\begin{array}{c} \Pi_{M}A_{1}{\Psi_{v}}^{*}\\ {A_{1}}^{*}\Psi_{v} \end{array}\right]}}R_{s}{H_{2}}^{T}$$

where $\Pi_{M}$ is used in Equation (7) to make the extended direction matrix $A_{1E}$ conjugate symmetric. Now the virtual aperture of subarray 1 is 2*M*, and its steering vector (i.e., the column of $A_{1E}$) can be expressed as:

(8) $$\begin{array}{l} a_{1E}(\theta_{k})\;=\\ {\left[e^{-j(M-1)N\pi \sin\theta_{k}}{v_{k}}^{*},e^{-j(M-2)N\pi \sin\theta_{k}}{v_{k}}^{*},...,{v_{k}}^{*},v_{k},v_{k}e^{jN\pi \sin\theta_{k}},...,v_{k}e^{j(M-1)N\pi \sin\theta_{k}}\right]}^{T} \end{array}$$

Due to the conjugate symmetric property, the steering vector in Equation (8) can also be transformed into a real-valued one, which is $h_{1}(\theta_{k})=Q_{2M}^{H}a_{1E}(\theta_{k})$. So the real-valued CCM with extended aperture is constructed:

(9) $$R_{h}=Q_{2M}^{H}R_{z}=H_{1}R_{s}{H_{2}}^{T}$$

where $H_{1}=\;[h_{1}(\theta_{1}),...,h_{1}(\theta_{K})]\;=\;Q_{2M}^{H}A_{1E}\in R^{2M\times K}$ is the extended real-valued direction matrix of subarray 1.

### 3.2. Ambiguous DOA Estimation

To obtain the signal subspaces of the two subarrays, SVD of the CCM in Equation (9) is performed:

(10) $$R_{h}=U\Lambda V^{T}$$

where U and V are left and right singular vectors, respectively. Λ is a *K* × *K* diagonal matrix composed of singular values. The real-valued signal subspaces U and V satisfy,

(11a) $$U=H_{1}T_{1}$$

(11b) $$V=H_{2}T_{2}$$

where $T_{1}$ and $T_{2}$ are two non-singular matrices.

Before the usage of the signal subspaces, we briefly review the properties of the direction matrices. Based on the Vandermonde structures of the direction matrices shown in Equations (2b) and (8), we define selecting matrices as $J_{1}=I_{2}⊗[0_{(M-1)\times 1},I_{\left(M-1\right)}]$, $J_{2}=I_{2}⊗[I_{\left(M-1\right)},0_{(M-1)\times 1}]$, $J_{3}=[I_{\left(N-1\right)},0_{(N-1)\times 1}]$, and $J_{4}=[0_{(N-1)\times 1},I_{\left(N-1\right)}]$, then the direction matrices satisfy:

(12a) $$J_{1}A_{1E}\Phi_{1}=J_{2}A_{1E}$$

(12b) $$J_{3}A_{2}\Phi_{2}=J_{4}A_{2}$$

where $\Phi_{1}=diag(e^{-jN\pi \sin\theta_{1}},...,$ $e^{-jN\pi \sin\theta_{2}})$ and $\Phi_{2}=diag(e^{-jM\pi \sin\theta_{1}},...,e^{-jM\pi \sin\theta_{2}})$ are diagonal matrices. As now the direction matrices are all transformed into real-valued ones ($H_{1}$ and $H_{2}$), then the relationships in Equation (12) are also transformed into real-valued forms [10]:

(13a) $$K_{1}H_{1}\Omega_{1}=K_{2}H_{1}$$

(13b) $$K_{3}H_{2}\Omega_{2}=K_{4}H_{2}$$

where K1=Re(Q(2M−1)HJ2Q2M), K2=Im(Q(2M−1)HJ2Q2M), K3=Re(Q(N−1)HJ4QN), and $K_{4}=$Im(Q(N−1)HJ4QN) are all real-values selecting matrices. $\Omega_{1}=diag(\tan(-N\pi \sin\theta_{1}/2),\cdots ,$ $\tan(-N\pi \sin\theta_{K}/2))$ and $\Omega_{2}=diag(\tan(-M\pi \sin\theta_{1}/2),\cdots ,)$ $\tan(-M\pi \sin\theta_{K}/2))$ are real-valued diagonal matrices.

Combine Equations (11) and (13), the signal subspaces satisfy:

(14a) $$K_{1}U\Sigma_{1}=K_{2}U$$

(14b) $$K_{3}V\Sigma_{2}=K_{4}V$$

where $\Sigma_{1}\;=\;T_{1}^{-1}\Omega_{1}T_{1}$ and $\Sigma_{2}\;=\;T_{2}^{-1}\Omega_{2}T_{2}$, which can be estimated via least squares (LS):

(15a) $$\Sigma_{1}={\left(K_{1}U\right)}^{+}K_{2}U$$

(15b) $$\Sigma_{2}={\left(K_{3}V\right)}^{+}K_{4}V$$

After Equation (15), the eigenvalues of $\Sigma_{1}$ and $\Sigma_{2}$ will, respectively, provide the estimations of the diagonal elements of $\Omega_{1}$ and $\Omega_{2}$, which can give the DOA estimations. However, as $\Sigma_{1}$ and $\Sigma_{2}$ are processed separately, and their eigenvalues are mis-pairing, which will cause the interference between the angles when determining the unique DOA. Sun et al. [20] proposed an additional processing method relying on peak search and angle difference chosen to deal with this problem, but this procedure also adds computation complexity. We will analyse the relationship between $\Sigma_{1}$ and $\Sigma_{2}$ below, and conduct a method to simultaneously obtain the eigenvalues of $\Sigma_{1}$ and $\Sigma_{2}$ with automatically pairing.

Firstly, substitute Equation (11) into Equation (10); then it can be obtained that $R_{h}=H_{1}T_{1}\Lambda {T_{2}}^{T}{H_{2}}^{T}$. Combine Equation (9), then it is shown that:

(16) $$T_{1}\Lambda {T_{2}}^{T}=\;R_{s}$$

Equation (16) means that $T_{2}=\;R_{s}{\left({T_{1}}^{T}\right)}^{-1}\Lambda^{-1}$, which can be substituted into $\Sigma_{2}=\;T_{2}^{-1}\Omega_{2}T_{2}$, then ${\Sigma_{2}}^{T}$ can be expressed as:

(17) $$\begin{array}{cc} {\Sigma_{2}}^{T} & =T_{2}^{T}\Omega_{2}{\left({T_{2}}^{-1}\right)}^{T}\\ & =\;\Lambda^{-1}{T_{1}}^{-1}R_{s}\Omega_{2}R_{s}T_{1}\Lambda \\ & =\;\Lambda^{-1}{T_{1}}^{-1}\Omega_{2}T_{1}\Lambda \end{array}$$

Combine $\Sigma_{1}\;=\;T_{1}^{-1}\Omega_{1}T_{1}$ and Equation (17), constructing a *K* × *K* matrix as:

(18) $$\begin{array}{cc} \Sigma & =\Sigma_{1}+j(\Lambda {\Sigma_{2}}^{T}\Lambda^{-1})\\ & =\;{T_{1}}^{-1}\Omega_{1}T_{1}+j{T_{1}}^{-1}\Omega_{2}T_{1}\\ & =\;{T_{1}}^{-1}(\Omega_{1}+j\Omega_{2})T_{1} \end{array}$$

where Λ has already been obtained after the SVD of CCM in Equation (10). According to Equation (18), the eigenvalues of Σ provide the estimations of the diagonal elements of $\Omega_{1}+j\Omega_{2}$, whose real part and imaginary part give the DOA information corresponding to subarray 1 and subarray 2, respectively. Use $\alpha_{k}$ to denote the *k*-th eigenvalue of Σ, then the DOA estimations from subarray 1 and subarray 2, respectively, are:

(19a) $$\sin{\overset{⌢}{\theta }}_{k,n}=-2\arctan(Re(\alpha_{k}))/(N\pi ),\;k=1,...,K$$

(19b) sinθ⌣k,m=−2arctan(Im(αk))/(Mπ), k=1,...,K

The DOA estimations from subarray 1 and subarray 2 are automatically paired based on the same $\alpha_{k}$. Due to the range limitation of the function arctan(⋅) ([−*π*/2, *π*/2]) in Equation (19), the two DOA estimations in Equation (19) may be ambiguous (original range within the tangent function is [−*πM*/2, *πM*/2] or [−*πN*/2, *πN*/2], which has been shown in $\Omega_{1}$ or $\Omega_{2}$ in Equation (13)), and unique DOA will be determined in the next section.

### 3.3. Unique DOA Estimation

Now two ambiguous DOA estimations are obtained from subarray 1 and subarray 2, respectively. In this section, we continue to uniquely determine the true DOA based on the coprime-ness between the two subarrays. As now the DOA estimations are automatically paired (corresponding to the same source), the angles can be determined without interference from other sources.

For the *k*-th DOA shown in Equation (19), due to the large inter-element spacing, there are totally *N* solutions for subarray 1 and *M* solutions for subarray 2, respectively [18,19,20]. The adjacent intervals between the estimations are 2/*N* for subarray 1 and 2/*M* for subarray 2, respectively:

(20a) $$\sin{\overset{⌢}{\theta }}_{k,n+1}-\sin{\overset{⌢}{\theta }}_{k,n}=\frac{2}{N},\;n=1,...,N-1$$

(20b) $$\sin{\overset{⌣}{\theta }}_{k,m+1}-\sin{\overset{⌣}{\theta }}_{k,m}=\frac{2}{M},\;m=1,...,M-1$$

Based on the relationship in Equation (20) and the obtained two arbitrary ambiguous estimations in Equation (19), all the *N* estimations $\sin{\overset{⌢}{\theta }}_{k,n},$ n=1,...,N for subarray 1 and all of the *M* estimations for subarray 2 $\sin{\overset{⌣}{\theta }}_{k,m},m=1,...,M$ can be obtained.

Finally, based on the coprime-ness between *M* and *N*, the unique estimation can be obtained by finding the coincidence between $\sin{\overset{⌢}{\theta }}_{k,n},\;n=1,...,N$ and $\sin{\overset{⌣}{\theta }}_{k,m},\;m=1,...,M$ [18]. In practical situations, the unique estimation is actually obtained from the average of two nearest ones:

(21) $${\hat{\theta }}_{k}=\arcsin(\frac{\sin{\overset{⌢}{\theta }}_{k,\hat{n}}+\sin{\overset{⌣}{\theta }}_{k,\hat{m}}}{2}),\;k=1,...,K$$

where $\sin{\overset{⌢}{\theta }}_{k,\hat{n}}$ and $\sin{\overset{⌣}{\theta }}_{k,\hat{m}}$ denote the two nearest results.

It should be noted that the CCM is estimated via finite snapshots in practice:

(22) $$R_{c}=\frac{1}{T}\sum_{t=1}^{T}\left(x_{1}(t)x_{2}^{H}(t\right))$$

where T denotes the number of snapshots. Therefore, the signal subspaces U and V are actually obtained by respectively selecting the left and right singular vectors corresponding to *K* largest singular-values.

### 3.4. Summaries and Remarks

The major steps of the proposed algorithm are:

1. Construct the real-valued CCM with extended aperture via Equations (22), (7), and (9).
2. Perform SVD of the CCM obtained in step 1 to obtain the signal subspaces, and estimate two initial ambiguous DOAs via Equations (15), (18), and (19).
3. Determine the unique DOA via Equations (20) and (21).

In our algorithm, the CCM construction, SVD and pseudo-inverse dominate the complexity, and it should be noted that the SVD and the pseudo-inverse are all based on real-valued computations, while the PSS method and ESPRIT based method both require multiple complex eigenvalue-decompositions and pseudo-inverses. The complexity of our algorithm is about *O*(*MNT* + *NM*^2^ + (2*M* + *N*)*K*^2^ + *K*^3^), which is lower than those of the PSS method and ESPRIT based method (PSS: *O*((*M*^2^ + *N*^2^)*T* + *M*^3^ + *N*^3^ + (*J*/*N*)*M*(*M* − *K*) + (*J*/*M*)*N*(*N* − *K*) and ESPRIT based: *O*((*M*^2^ + *N*^2^)*T* + *M*^3^ + *N*^3^ + 3(*M* + *N*)*K*^2^ + 4*K*^3^) without considering the additional peak search based processing procedure).

The advantages of our algorithm can be summarized as:

1. It requires CCM construction, real-valued SVD, and eigenvalue-decomposition only once, so it has low complexity.
2. It extends the aperture of subarray 1, thus, the number of managed source is increased (according to Equation (15), the maximum number depends on the minimum aperture between the two subarrays, i.e., min (2*M* − 1, *N* − 1)).
3. It achieves better DOA estimation performance than the PSS method and the ESPRIT-based method.

The last two advantages will be verified in the simulation section below.

**Remark** **1.** *For the situation when M > N, we should consider to extend the aperture of subarray 2 to increase source number that the method can handle. Just transform the direction matrix of subarray 1 into real-valued one firstly:*

(23) $$\begin{array}{cc} R_{cr2} & =Q_{M}^{H}R_{c}=Q_{M}^{H}A_{1}R_{s}A_{2}^{H}\\ & =G_{1}\Psi_{u}R_{s}A_{2}^{H} \end{array}$$

*where* $G_{1}$ *is the real-valued direction matrix and* $\Psi_{u}$ *is the diagonal matrix composed of the rest scalars (similar to the processing of subarray 2 in Equation (4)). Then construct the extended CCM as:*

(24) $$\begin{array}{cc} R_{z} & =[R_{cr2},{R_{cr2}}^{*}]\\ & =[G_{1}\Psi_{u}R_{s}A_{2}^{H},G_{1}{\Psi_{u}}^{*}R_{s}A_{2}^{T}]\\ & =G_{1}R_{s}{\left[A_{2}{\Psi_{u}}^{*},{A_{2}}^{*}\Psi_{u}\right]}^{H} \end{array}$$

*Now the aperture of subarray 2 has been extended, and similar steps as those from Equation (8) can be conducted.*

**Remark** **2.** *In real situations, the residual noise will make* $R_{h}$ *in Equation (9) none real-valued, so the real-valued CCM is actually acquired via* Rh=Re(Q2MHRz).

## 4. Simulation Results

Consider coprime arrays with *M* = 5 antennas for subarray 1 and *N* = 7 antennas for subarray 2, respectively. Assume there are two uncorrelated signals with DOAs being $\theta_{1}=15^{\circ }$ and $\theta_{2}=30^{\circ }$, respectively. Collect *T* = 200 snapshots, and define the root mean square error (*RMSE*) of the DOA estimation as:

(25) $$RMSE=\frac{1}{K}\sum_{k=1}^{K}\sqrt{\frac{1}{L}\sum_{l=1}^{L}\left[{\left({\hat{\theta }}_{k,l}-\theta_{k}\right)}^{2}\right]}$$

where ${\hat{\theta }}_{k,l}$ is the estimation of $\theta_{k}$ of the *l*-th Monte Carlo trial, and the total trial number is *L* = 200. In the simulations below, the DOA estimation performance comparison between the PSS method [19], ESPRIT based method [20] and the proposed algorithm under the measurement of RMSE will be presented. The PSS method uses search grid 0.1°, and both PSS method and ESPRIT based method exploit additional peak search based procedure [20] to avoid the interference between sources after obtaining the DOA estimations. Multi-source Cramer-Rao Bound (CRB) [23] of the DOA estimation using coprime arrays is also presented as a benchmark.

**Figure 2:**
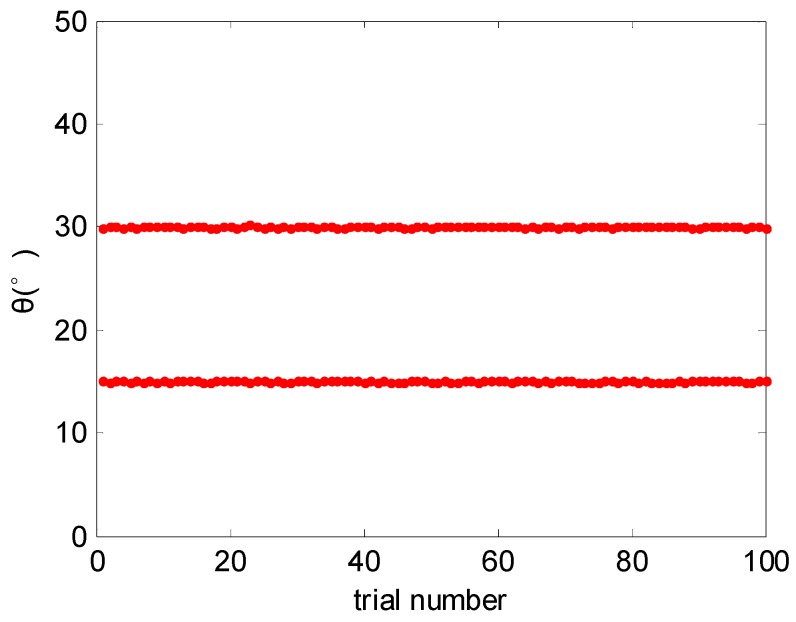
DOA estimation results over 100 trials (SNR = 0 dB).

Figure 2 shows the DOA estimation results of the proposed algorithm over 100 trials when SNR = 0 dB, and it is shown that the proposed algorithm can accurately estimate both of the two DOAs.

**Figure 3:**
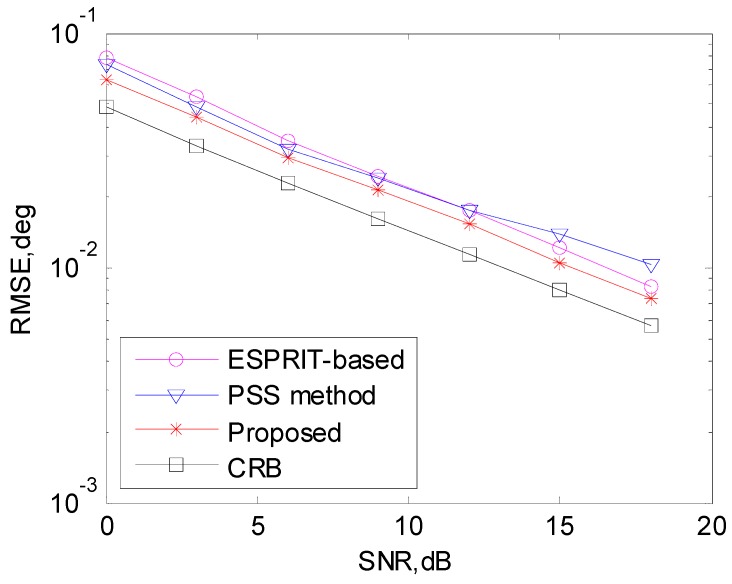
DOA estimation performance comparison.

The DOA estimation performance comparison is shown in Figure 3, and it is indicated that our algorithm has better DOA estimation performance than the PSS method and the ESPRIT-based method due to the extended aperture and automatically solutions from the two subarrays. Due to the self-limitation of peak search based method [20], the PSS method performs worse than the ESPRIT-based method with high SNR.

**Figure 4:**
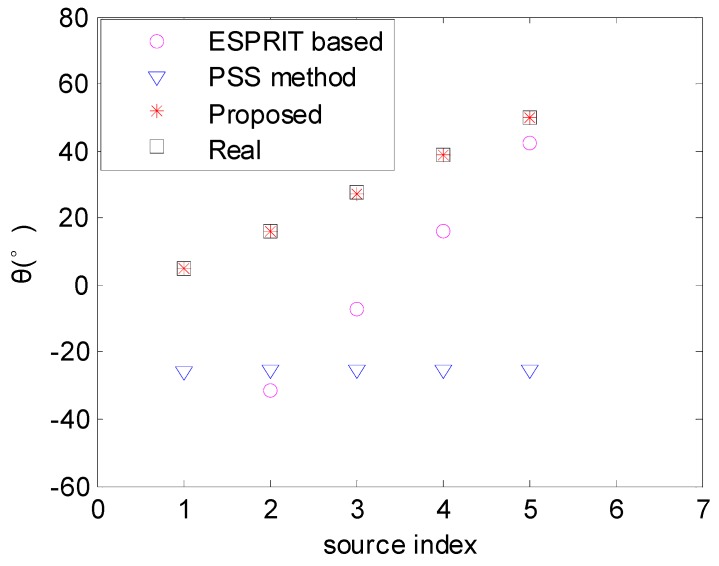
DOA estimation results with *K* = 5 sources (SNR = 10 dB).

Figure 4 shows the DOA estimation results when the source number is *K* = 5, and the angles of the sources are uniformly distributed among the range [5°, 50°]. As *M* < *N*, the maximum source number that the PSS method and the ESPRIT-based method can handle depends on the minimum aperture between the two subarrays, i.e., (*M* − 1) = 4. In contrast, the proposed algorithm can deal with min(2*M* − 1, *N* − 1) = 6 sources due to the aperture extension in Section 3.1. Thus, Figure 4 verifies that the proposed algorithm can handle more sources than the PSS method and the ESPRIT-based method.

**Figure 5:**
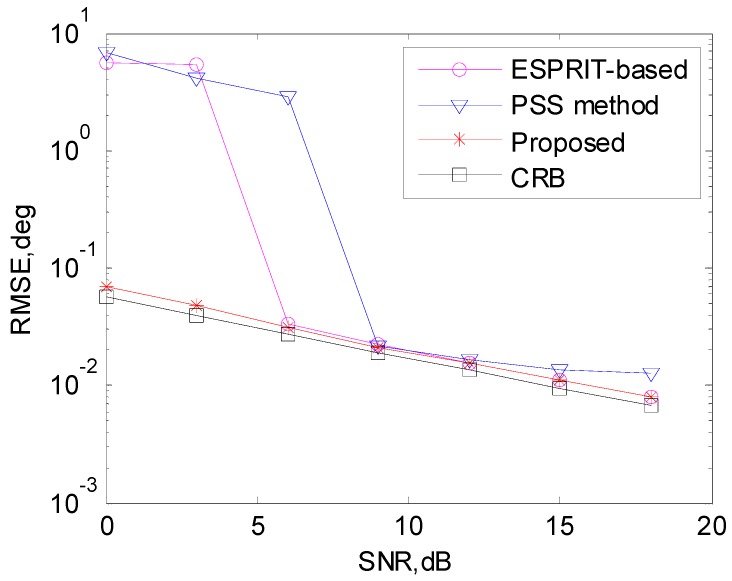
DOA estimation performance comparison with closely-spaced sources.

In Figure 5, we show the DOA estimation performance comparison with two closely-spaced sources, whose DOAs are $\theta_{1}=12^{\circ }$ and $\theta_{2}=15^{\circ }$, respectively. It is shown that both the PSS method and the ESPRIT-based method have significant performance degradations with low SNR compared to those shown in Figure 3, while the proposed method still maintains stable performance. With high SNR, their estimation errors are close to each other due to the closely-spaced sources.

**Figure 6:**
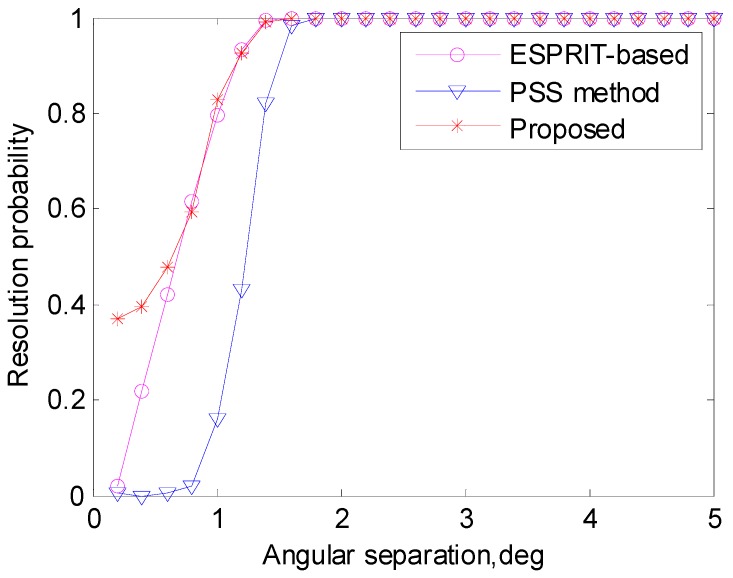
Resolution probability versus angular separation (SNR = 10 dB).

To clearly observe the resolutions of the algorithms, we use resolution probability to investigate the DOA estimation performance versus angular separation in Figure 6. The two DOAs are $\theta_{1}=50^{\circ }$ and $\theta_{2}=\theta_{1}+\Delta \theta$, where Δθ denotes the angular separation. The two sources can be resolvable if both $\left|{\hat{\theta }}_{1}-\theta_{1}\right|$ and $\left|{\hat{\theta }}_{2}-\theta_{2}\right|$ are smaller than $\left|\theta_{1}-\theta_{2}\right|/2$ [20]. The SNR is set to 10 dB, and it is shown that the proposed algorithm achieves the best resolution performance among the algorithms. 

**Figure 7:**
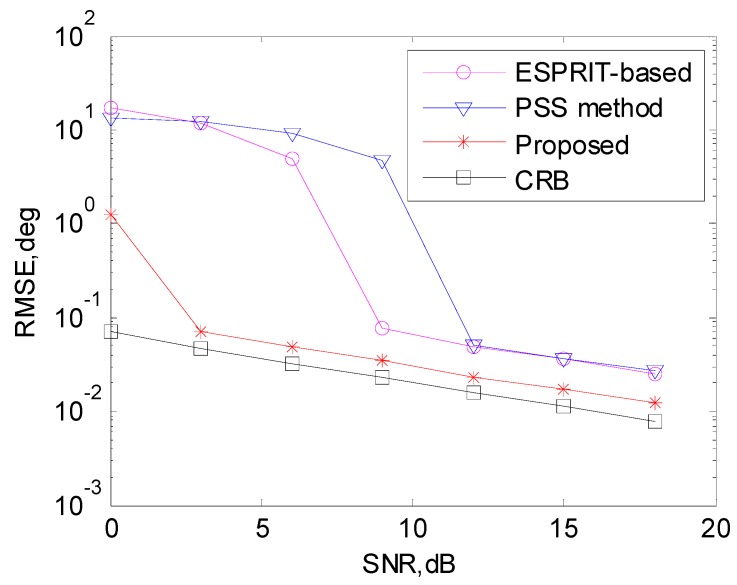
DOA estimation performance comparison with three sources.

Figure 7 shows the DOA estimation performance comparison with *K* = 3 sources, whose DOAs are 30°, 35°, and 50°, respectively. With the increase of the source number, it is indicated that both PSS method and the ESPRIT-based method degrade significantly, especially with low SNR. The proposed algorithm still outperforms the other methods and also has performance degradation with low SNR compared to the two-source situation.

## 5. Conclusions

A real-valued CCM based fast DOA estimation method for coprime arrays is proposed. A real-valued CCM with extended aperture is constructed and is then exploited to obtain the closed-form solutions of DOA estimations from the two subarrays. By analysing the relationship between the two subarrays, the obtained solutions are automatically paired, which can avoid angular interference when determining the unique DOA estimation. Compared to the PSS method and the ESPRIT-based method for coprime arrays, the proposed algorithm reduces the computational burden, achieves better DOA estimation performance, and handles more sources. Several simulations have been carried out to verify the validity of our algorithm.
